# Usefulness of vacuum-assisted closure after stoma closure with purse string suturing: a retrospective trial

**DOI:** 10.20407/fmj.2021-007

**Published:** 2021-11-25

**Authors:** Koji Masumori, Kotaro Maeda, Tsunekazu Hanai, Harunobu Sato, Yoshikazu Koide, Hiroshi Matsuoka, Hidetoshi Katsuno, Tomoyoshi Endo, Yeongcheol Cheong, Ichiro Uyama

**Affiliations:** 1 Department of Gastroenterological Surgery, Fujita Health University, School of Medicine, Toyoake, Aichi, Japan; 2 International Medical Center, Fujita Health University Hospital, Toyoake, Aichi, Japan; 3 Department of Gastroenterological Surgery, Fujita Health University Okazaki Medical Center, Okazaki, Aichi, Japan

**Keywords:** Vacuum-assisted closure, Purse string suture, Stoma closure

## Abstract

**Objectives:**

Surgical site infection (SSI) is a problematic complication after stoma closure. The purse string suture (PSS) technique eliminates this problem, but the area takes longer to heal. The present retrospective study was performed to evaluate the usefulness of a vacuum-assisted closure (VAC) system for the promotion of wound healing after stoma closure.

**Methods:**

Consecutive patients undergoing stoma closure with the PSS technique were divided into two groups: those treated with and without use of the VAC system. The volume of dead space and the size of the wound were measured after stoma closure in both groups. The same measurements were performed on days 3 and 7 after closure. The time needed for wound closure was also examined in both groups. Outcomes were also evaluated according to age, body mass index, operative time, bleeding volume, wound consistency, patient satisfaction, perioperative inflammatory response, occurrence of SSI, and hospitalization days.

**Results:**

The VAC group comprised 31 patients, and the non-VAC group comprised 34 patients. The volume of dead space on days 3 and 7 after closure was significantly smaller in the VAC group than in the non-VAC group (P=0.006 and P<0.001, respectively). The number of SSIs was significantly lower in the VAC group than in the non-VAC group (P=0.014).

**Conclusion:**

The dead space volume on days 3 and 7 after stoma closure with PSS significantly decreased by using the VAC system. The incidence of SSI after stoma closure also significantly decreased by using the VAC system.

## Introduction

A high complication rate of 1.2% to 40.0% has been reported after stoma closure.^1–8^ Such complications include wound infection, bleeding, anastomotic stenosis, and leakage, with wound infection being the most common. The reported wound infection rates after ileostomy and colostomy closure range from 0% to 18%^4,6,7^ and from 1.2% to 3.0%,^5,8^ respectively. In patients undergoing colostomy, surgical site infection (SSI) caused by *Escherichia coli* can easily occur after stoma closure. Because SSI has been found to cause increases in wound healing and hospitalization times, creation of an ileostomy has been the preferred method of a diverting stoma. However, an ileostomy causes dehydration and renal dysfunction due to electrolyte abnormalities, high output,^9^ and outlet obstruction,^10,11^ eventually resulting in weight loss and discontinuation of chemotherapy. In contrast, a colostomy is beneficial for stoma care because of hard stool discharge. We have adapted the colostomy to serve as a diversion of stool except in patients with adhesion or a fatty transverse colon. To decrease the incidence of SSI after colostomy closure, the purse string suture (PSS) technique^12–16^ has been used.^2,5,6^ Although the PSS technique can decrease the incidence of SSI, this technique basically involves tertiary healing. The burden on the patient is heavy because of the unclean wound resulting from serous discharge, necessitating daily gauze changes. Therefore, we considered that negative-pressure wound therapy (NPWT)^17–22^ using a vacuum-assisted closure (VAC) system would help to solve this problem because of its positive effect on granulation growth. To test this hypothesis, a retrospective study was carried out to evaluate the usefulness of the VAC system for promoting wound healing and decreasing SSI.

## Methods

From 2014 to 2018, 175 patients underwent stoma closure surgery at Fujita Health University. Among them, 31 patients who underwent the PSS technique accompanied by use of the VAC system (VAC group) and 34 patients who underwent the PSS technique without use of the VAC system (non-VAC group) were enrolled in the study. We collected the patients’ clinical background data, age, sex, body mass index (BMI), colostomy or ileostomy, anastomosis method, underlying disease, comorbidities, dead space volume, wound size, dead space reduction rate, time until stoma closure, operative time, bleeding, biochemical indices, length of hospital stay, and incidence of SSI. These data were then correlated with each procedure and the postoperative hospital stay and statistically evaluated. SSI was defined as infection that occurred within 30 days after surgery and was characterized by purulent drainage, tenderness, redness, and localized swelling. The wound condition of the patients who were discharged within 30 days was checked by outpatient doctors between 28 and 35 days after surgery.

### Technique

**Figure 1:**
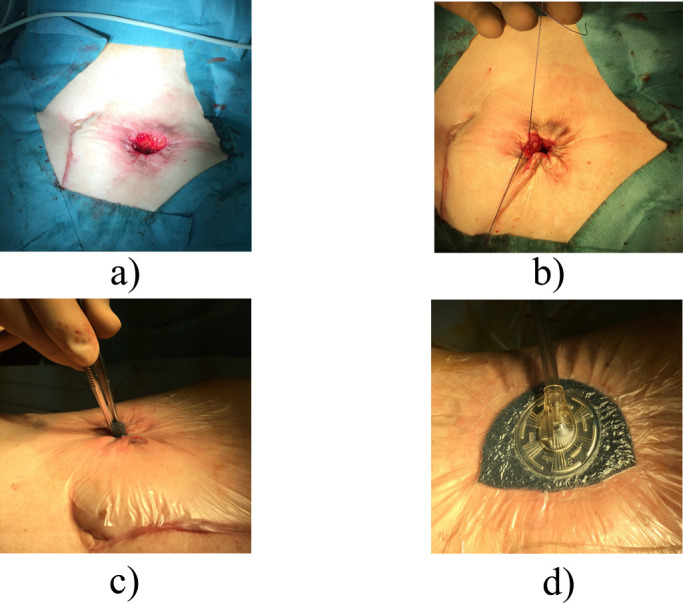
(a) Skin incision after stoma closure. A skin incision was performed 4 mm from the mucocutaneous region to prepare a circle shape. Even when the stoma was oval-shaped, the skin incision was performed to create a circle shape. (b) Purse string suture (PSS) technique. The dermis was sutured at 5-mm intervals using 2-0 Vicryl absorption yarn. The suture was tied to prepare an approximately 1-cm drainage hole. (c) A small piece of Granufoam was inserted into the drainage hole. (d) VAC system application. A suction tube was attached to the drape. The suction pressure was maintained at –125 mmHg. In case of any air leakage, an additional drape was added.

At the time of stoma closure, the stoma opening was closed by suturing to minimize contamination. A skin incision was performed 4 mm from the mucocutaneous region to form a circular shape. Even when the stoma was oval-shaped, the skin incision was performed to form a circular shape. A Gambee or Albert-Lembert anastomosis was performed using 4-0 polydioxanone after dissecting the intestinal adhesion with trimming of the stoma orifice. The re-anastomosed intestine was returned to the abdomen, the fascial layer was sutured, and the surgical site was sterilized with 100 mL of saline. If tightening of the skin occurred after performing the PSS technique, the subcutaneous layer was dissected by electrocautery (Figure 1a).

### PSS technique

The dermis was sutured at 5-mm intervals using 2-0 Vicryl absorption yarn. The suture was tied to create an approximately 1-cm drainage hole. To prevent early closure of the wound, the drainage hole was packed with gauze, which was replaced daily for 3 days after surgery. The patient was thereafter instructed to wash the drainage hole by shower. The threads were not removed (Figure 1b). Antibiotics were given for 1 to 3 days after surgery.

### VAC technique

After completion of the PSS technique, a 19-×13-cm drape was attached around the drainage hole for VAC therapy. The drape around the hole of the stoma closure site was cut to create a drainage hole. A small piece of Granufoam was inserted into the drainage hole (Figure 1c), and a medium-sized piece of Granufoam was then added. These were covered by the drape. The center of the drape covering was cut to 1.5 cm in diameter. Finally, a suction tube was attached to the drape (Figure 1d). The suction pressure was maintained at –125 mmHg. In case of any air leakage, an additional drape was added. The VAC system was replaced on day 3 after surgery and removed on day 7.

### Measurement of dead space

The dead space of the wound was filled with saline to measure its volume on days 3 and 7 postoperatively. The major axis of the hole was also measured.

### Statistical analysis

Data are expressed as median (range). Categorical data are expressed as the count number. Pearson’s chi-square test or Fisher’s exact test with Yates correction was used to compare differences in categorical variables where appropriate. For continuous variables, two-group comparisons were carried out using the Mann–Whitney U test. Logistic regression analysis was used for multivariate analysis. SPSS version 19 (SPSS Japan Inc., Tokyo, Japan) was used to conduct the statistical analyses. A two-tailed P value of <0.05 was considered statistically significant.

## Results

The patients’ characteristics are shown in Table 1. There were no significant differences in age, sex, BMI, colostomy/ileostomy, anastomosis method (Gambee/Albert-Lembert), underlying disease, or comorbidities between the VAC group and the non-VAC group (Table 1). There were also no significant differences in the time period from stoma construction to stoma closure, operative time, bleeding volume, or postoperative inflammation between the two groups (Table 2).

Although the dead space on the day of the operation was equivalent in both groups, it was significantly less in the VAC group on day 3 (P=0.006) and day 7 (P=0.001) (Table 3). The area of the stoma hole was significantly smaller in the VAC group than in the non-VAC group on day 7 (P=0.016) (Table 3), although the area had been equivalent between the two groups on the day of the operation.

The reduction rate of the dead space volume from day 0 to 3 was 60.0% in the VAC group and 46.7% in the non-VAC group, and the difference was statistically significant (Table 4). The reduction rate of the dead space volume from day 0 to 7 and from day 3 to 7 was also significantly different between the two groups (Table 4). There was no significant difference in postoperative complications other than SSI between the two groups. Notably, the incidence of SSI was significantly lower in the VAC group than in the non-VAC group (P=0.014) (Table 5). There was no significant difference in the length of hospital stay between the two groups (Table 5).

## Discussion

**Figure 2:**
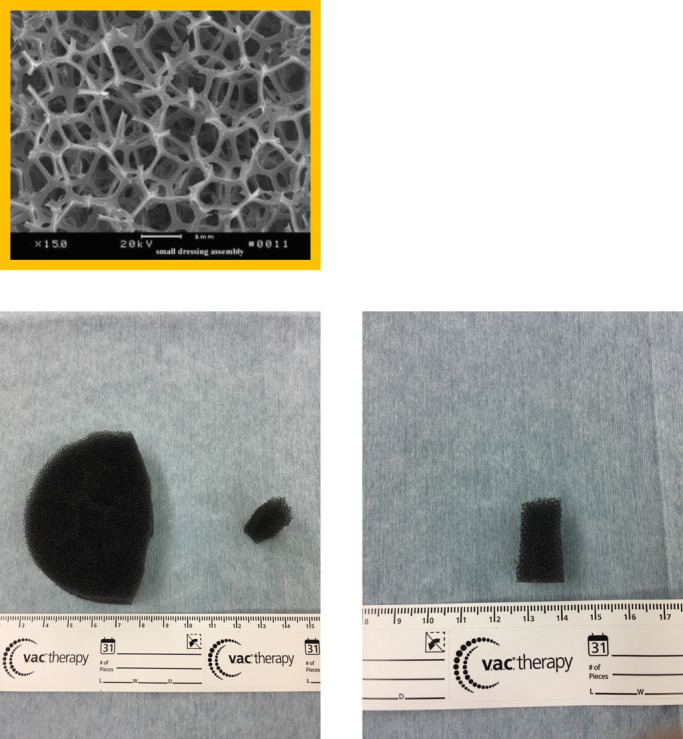
Granufoam (small and medium-sized). Granufoam is made of polyurethane. It has a network structure and hydrophobicity, which stimulates granulation tissue formation and contraction of the wound’s edge. A small piece of Granufoam is also considered to be effective for the drainage hole of the PSS technique because it allows drainage from deep within the dead space. a medium-sized piece of Granufoam is placed on the small piece of Granufoam, and there is on the surface of the skin. Finally, the VAC system is applied and negative pressure is begun.

We performed simple closure for most patients in the early period of this study, resulting in a high incidence of wound infection; therefore, we performed the PSS technique in the later period. However, because serous discharge occurred even after use of the PSS technique, NPWT (VAC system) was added to the PSS closure in the latest period. Bias may have been present in the times for each population of this study, as in historical control studies. NPWT has been used to treat many diseases, including fistulas,^19^ decubitus ulcers, open fractures,^20^ and abdominal trauma,^21^ to eliminate the dead space and remove the exudative fluid and waste products by applying sustained or intermittent negative pressure. By further protecting the surface, NPWT is considered to be more effective in preventing infection, promoting granulation growth, inducing vascular development, reducing the area of dead space, and preventing the growth of bacteria. When infection occurs after stoma closure, the hospitalization period is extended and the patient’s quality of life is decreased. We introduced the VAC system after stoma closure along with the PSS technique to reduce the incidence of infection and the hospital stay. Before introducing the PSS technique, we performed a simple sewing procedure; however, the infection rate was quite significant. Banerjee^23^ reported a 40% infection rate after application of simple sutures, and Vermulst et al.^24^ found that the infection rate after ileostomy closure was 36%. Many reports have indicated that the infection rate decreases with the PSS technique when compared with simple sutures.^12–14,25–31^ Therefore, we adopted the PSS technique, but SSI sometimes still occurred. Therefore, we added the VAC system to the PSS technique to reduce infection. Fleming and Gillen^32^ reported that a longer interval between the closure of the first operation and laparoscopic Hartmann surgery (>9 months) was associated with a higher risk of postoperative complications. Tang et al.^33^ reported that any operation exceeding 3 hours is a risk factor for wound infection. Hu et al.^8^ reported that the use of silk yarn reduced the surgical time and wound infection rate. Rheumatoid arthritis, liver cirrhosis, steroid use,^32^ the detrimental effects of steroid dependence and preoperative hypoalbuminemia,^34^ and American Society of Anesthesiologists scores of >3^31^ reportedly cause delayed healing and infection. In the present study, there was no significant difference in the operation time and the period until closure of the stoma and comorbidity between the two groups (P=0.916 and P=0.213, respectively). The volume of dead space was measured on the day of surgery and on days 3 and 7 after surgery to study the promoting effect of our technique on wound healing. The volume of dead space on days 3 and 7 was significantly smaller in the VAC group (P=0.006 and P<0.001, respectively; Mann–Whitney test). We found that using the VAC system, inserting a small piece of Granufoam into the dead space, and adding negative pressure promoted growth of granulation tissue and vascular development. Granufoam is made of polyurethane. It has a network structure and hydrophobicity, which stimulates the formation of granulation tissue and contraction of the wound’s edge. Granufoam is also considered to be effective for drainage of the PSS site because it allows drainage from deep within the dead space (Figure 2). As a result, the infection rate was significantly lower in the VAC group (P=0.014). We considered that the suction pressure, set at –125 mmHg, induced the growth and contraction of granulation tissue. The proper suction pressure varies among different institutes. A unified view concerning suction pressure has not yet been established. Morykwas et al.^17^ and Argenta and Morykwas^18^ studied laser Doppler-measured blood flow in the wound and adjacent tissue, the rate of granulation tissue formation, and the clearance of bacteria from infected wounds with regard to suction pressure using pig models. The blood flow levels increased fourfold when an atmospheric pressure of less than –125 mmHg was applied. A significant increase in the rate of granulation tissue formation (P≤0.05) reportedly occurred at both continuous (63.3%±26.1%) and intermittent (103%±35.3%) pressure settings. Isago et al.^35^ reported that even after the suction pressure was lowered by at least −50 mmHg, there was no significant difference in the shrinkage rate because of its pressure. Patients complained of pain at a pressure of –125 mmHg. In our hospital, the VAC system was set to a suction pressure of –125 mmHg. If patients complained of pain, analgesics were administered. If we had found that there was no burden on the patient and no significant difference in the shrinkage rate even after the setting was lowered, we might have considered further researching the suction pressure setting. With respect to the hospitalization period, a shorter hospital stay was expected in the VAC group because of the promotion of granulation growth. However, no significant reduction was noted (P=0.659). This might have been because many patients opted to extend their hospitalization period even when the postoperative course was uneventful. Sureshkumar et al.^26^ defined the duration of wound healing as the time required for nearly complete epithelialization without any discharge or SSI. Camacho-Mauries et al.^14^ examined the healing period after linear closure versus the PSS technique. The healing time was significantly different between the two groups: 5 to 9 weeks in the linear closure group and 3 to 8 weeks in the PSS group (P<0.0001). However, the authors did not provide a definition of epithelialization. Although the authors had initially planned to examine the healing period under the epithelium, this analysis was excluded because the definition of epithelialization was unclear, and different outpatients’ doctors had to decide on the term. Formation of an incisional hernia^13,14,28^ has been confirmed during the follow-up period after stoma closure. No incisional hernias occurred after stoma closure in the present study.

A new type of NPWT, the V.A.C. ULTA therapeutic system with the function of washing the VAC therapy site, is available for purchase; it is thought to promote wound healing. Additionally, because of its ability to maintain a wetter state, it is also thought to promote the healing of contaminated wounds, locally infected wounds, and intractable infections. We are considering a future study involving comparison with VAC therapy.

## Conclusion

The occurrence of SSI was clearly lower in the VAC group than in the non-VAC group. However, there was no decrease in the period of hospitalization or the period up to the epithelium.

## Figures and Tables

**Table1:** Patients’ characteristics

	VAC group (n=31)		Non VAC group (n=34)		*P-value*
Age (years)	67.0 (24–90)		61.5 (24–84)		0.086
Male/Female	19/12		24/10		0.446
BMI	22.7 (13.5–40.2)		21.6 (16.5–38.5)		0.382
colostomy/ileostomy	25/6		27/7		1.000
Gambee/AL anstomosis	30/1		32/2		1.000
Underlying disease (malignancy/benign)	Rectal cancer	17	Rectal cancer	23	1.000
	Ulcerative colitis	2	Ulcerative colitis	2	
	Perforation of appendicitis	1	Sigmoid colon cancer	1	
	Perforation of diverticulitis of the colon	3	Perforation of diverticulitis of the colon and ileum	4	
	Iatrogenic perforation	1	Ileus	2	
	Birth canal laceration	1	Familial Poliposis Coli	1	
	Anastomotic leak	2	Sigmoid colon cancer	1	
	Sigmoid colon cancer	3			
	Torsion of Sigmoid colon	1			
Comorbidity	Cardiovascular disease	1	Renal Failure (dialysis)	1	0.095
	Rheumatoid arthritis	2			
	Using steroid	2			
	Diabetes mellitus	1			
	Liver cirrhosis	1			

Clinical background data of the VAC group and non-VAC group. There were no significant differences in age, BMI, colostomy/ileostomy, anastomosis method (Gambee/Albert-Lembert), underlying diseases, or comorbidities between the VAC group and the non-VAC group.

**Table2:** Demographic and operative outcome data of the VAC group and non VAC group

	VAC group (n=31)	Non VAC group (n=34)	*P-value*
Period to stoma closure (M)	10 (3–40)	10 (5–39)	0.213
Operative time (min)	97 (59–246)	96 (45–320)	0.916
Bleeding (ml)	24 (0–239)	23 (0–342)	0.782
Postoperative day 1 WBC	9400 (5800–24600)	10700 (3900–20500)	0.321
Postoperative day 3 WBC	7300 (3300–12900)	7300 (4700–10800)	0.641
Postoperative day 7 WBC	5700 (3500–11100)	6150 (2600–9800)	0.906
Postoperative day 1 CRP	4.2 (0.4–12.0)	4.7 (0.6–15.6)	0.306
Postoperative day 3 CRP	6.3 (0.3–19.7)	4.9 (0.0–22.6)	0.358
Postoperative day 7 CRP	1.3 (0–7.0)	1.0 (0–26.9)	0.389

The surgical treatment, period until stoma closure, operative time, and bleeding were compared with regard to the amount of dead space on the day of surgery. There was no significant difference in the white blood cell count or C-reactive protein concentration on day 1, 3, or 7 between the VAC group and the non-VAC group.

**Table3:** Dead space volume and wound size

	VAC group (n=31)	Non VAC group (n=34)	*P-value*
Dead space on the day of operation (ml)	1.0 (0.4–20.0)	1.5 (0.2–8.0)	0.207
on 3rd postoperative day (ml)	0.4 (0.1–2.0)	0.8 (0.1–2.2)	0.006
on 7th postoperative day (ml)	0.1 (0–0.6)	0.6 (0–2.4)	<0.001
Stoma hole area on the day of operation (cm^2^)	1.5 (0.7–5.0)	2.0 (0.6–7.3)	0.178
Stoma hole area on 7th postoperative day (cm^2^)	1.0 (0.1–10.5)	1.3 (0.2–6.7)	0.016

Although the dead space was equivalent in both groups on the day of the operation, it was significantly different on day 7 (P<0.001).

**Table4:** Reduction rate of dead space of stoma hole

Reduction rate of the dead space of the stoma hole	VAC group (n=31)	Non VAC group (n=34)	*P-value*
0 postoperative day/3 postoperative day (%)	–60 (max-98.5min250)	–46.7 (max-95.0min60.0)	0.028
0 postoperative day/7 postoperative day (%)	–90.4 (max-100.0min25.0)	–65.5 (max-100.0min43.3)	0.001
3 postoperative day/7 postoperative day (%)	–71.4 (max-100.0min100)	–28.2 (max-100.0min93.6)	0.001

The reduction rate of the dead space volume from day 0 to 3 was 60.0% in the VAC group and 46.7% in the non-VAC group with a statistically significant difference.

**Table5:** Postoperative complications

	VAC group (n=31)	Non VAC group (n=34)	*P-value*
Surgical site infection (SSI)	1/31	9/34	0.014
Complication after operation except SSI	2/31	2/34	1.000
Length of hospital stay (days)	11.0 (8.0–30.0)	12.0 (7.0–41.0)	0.544

There was no significant difference in postoperative complications other than SSI between the two groups. Notably, the incidence of SSI was significantly lower in the VAC group than in the non-VAC group. There was no significant difference in the length of hospital stay between the two groups (11 vs. 12 days).
